# Histone Acetyltransferase (HAT) P300/CBP Inhibitors Induce Synthetic Lethality in PTEN-Deficient Colorectal Cancer Cells through Destabilizing AKT

**DOI:** 10.7150/ijbs.42197

**Published:** 2020-03-25

**Authors:** Yifan Liu, Eun Ju Yang, Changxiang Shi, Pui Kei Mou, Baoyuan Zhang, Changjie Wu, Junfang Lyu, Joong Sup Shim

**Affiliations:** Cancer Centre, Faculty of Health Sciences, University of Macau, Taipa, 999078, Macau

**Keywords:** PTEN, Synthetic lethality, Histone acetyltransferases, Anacardic acid, AKT, Hsp70

## Abstract

PTEN, a tumor suppressor, is found loss of function in many cancers, including colorectal cancer. To identify the synthetic lethal compounds working with PTEN deficiency, we performed a synthetic lethality drug screening with PTEN-isogenic colorectal cancer cells. From the screening, we found that *PTEN^-/-^* colorectal cancer cells were sensitive to anacardic acid, a p300/CBP histone acetyltransferase (HAT) inhibitor. Anacardic acid significantly reduced the viability of *PTEN^-/-^* cells not in *PTEN^+/+^* cells via inducing apoptosis. Inhibition of HAT activity of p300/CBP by anacardic acid reduced the acetylation of histones at the promoter region and inhibited the transcription of Hsp70 family of proteins. The down-regulation of Hsp70 family proteins led to the reduction of AKT-Hsp70 complex formation, AKT destabilization and decreased the level of phosphorylated AKT at Ser473, all of which are vital for the survival of *PTEN^-/-^* colorectal cells. The synthetic lethality effect of anacardic acid was further validated in tumor xenograft mice models, where *PTEN^-/-^* colorectal tumors showed greater sensitivity to anacardic acid treatment than *PTEN^+/+^* tumors. These data suggest that anacardic acid induced synthetic lethality by inhibiting HAT activity of p300/CBP, thereby reducing Hsp70 transcription and destabilizing AKT in PTEN deficient colorectal cancer cells.

## Introduction

Phosphatase and tensin homolog (PTEN), a negative regulator of AKT, is a key tumor suppressor gene, which are found mutated or deleted in many cancers. Tumor suppressor genes are the safeguards of regulations of cell cycle, cell adhesion, cell proliferation, cell migration, cell death and DNA damage repairs 1. Mutation or deletion of tumor suppressor genes is considered as loss of function and lead to functional disruption of these genes by losing inhibition to tumor development 1. Loss-of-function of PTEN can also be gained from epigenetic silencing. Hypermethylation of CpG islands of PTEN promoter which leads to PTEN transcription silence is found in colorectal, breast, prostate cancer and other cancers 2-4. The importance of PTEN makes it become a vital target for cancer therapy.

Based on the strategy of synthetic lethality, taken mutation or deletion of tumor suppressor genes as one part in synthetic lethality system, it is prospective to screen partners working in combination with these genes to kill specific tumor suppressor deficient cancer cells. As PTEN is often found mutated or deleted in colorectal cancer CRC 2, 5, 6, synthetic lethality approach targeting *PTEN^-/-^* cancer may provide new treatment opportunities for such cancer types 7. To identify drugs that are selective for *PTEN^-/-^* CRC cells, a synthetic lethality drug screening with PTEN-isogenic CRC cell line pair was carried out. Anacardic acid (AA) was identified as a synthetic lethality partner of PTEN deficiency in the screening. AA, a salicylic acid derivative, was a natural HAT inhibitor extracted from Cashew nut shell liquid 8. AA was reported to inhibit the HAT activity of p300 and p300/CBP-associated factor (PCAF) 9. It also has been suggested to have anti-microbial, anti- inflammatory and anti-tumor activities in previous research 10-13. Recent studies suggested that AA could sensitize prostate cancer to ion-radiation (IR) in the androgen receptor dependent pathway 14, 15. AA induced cell apoptosis through autophagy by ER stress and suppressing DAPK3/ AKT/m-TOR signaling pathway in prostate cancer 16. However, the anti-cancer activities of AA and its relationship with different PTEN status are unclear in CRC. We here report that AA induces cell apoptosis in a synthetic lethal way in *PTEN^-/-^* CRC cells. Mechanistic exploration shows the involvement of HAT-Hsp70- AKT pathway in the AA-induced synthetic lethality in *PTEN^-/-^* CRC cells.

## Materials and Methods

### Cell culture and reagents

The colorectal cancer cell line: HCT116 cells and the prostate cancer cell lines: PC3 and 22RV1 cells were purchased from American Type Tissue Collection (ATCC, Manassas, VA, USA). They were cultured with RPMI-1640 supplemented with 10% Fetal Bovine Serum (#26140079, Thermo Fisher Scientific, Waltham, MA, USA) and 1% penicillin and streptomycin (#15140163, Thermo Fisher Scientific) at 37° of CO_2_ incubator. The kinase inhibitor drug library (#L1200, Selleck), Anacardic acid, C646, cycloheximide (CHX), MG132 and MKT077 were purchased from Selleck Chemicals (Houston, TX, USA).

### Synthetic lethality screening

The synthetic lethality screening was carried out with *PTEN^+/+^* and *PTEN^-/-^* HCT116 isogenic cells in 384-well plate format following the established procedure 17. A kinase inhibitor library containing 430 small molecules were used for synthetic lethality screening.

### Immunoblotting and antibodies

Cells were treated with RIPA buffer (20 mM Tris pH 7.6, 150 mM NaCl, 1% NP40) combined with cOmplete protease inhibitor cocktail (#06538282001, Roche, West Sussex, UK) and lysed for immunoblots. Immunoblots were carried out with 15% and 6% SDS-PAGE gels based on the molecular weight of proteins. The primary antibodies used were listed in Supplementary Table S1. The anti-rabbit and anti-mouse secondary antibodies conjugated with HRP (horse radish peroxidase) were purchased from Santa Cruz and life technology. Western blot signals were detected using Clarity™ Western ECL Substrate (Bio-Rad, Hercules, CA) under a Bio-Rad ChemiDoc MP imaging system.

### RNA interferences (RNAi)

The shRNAs of p300 and CBP (#TG313197) were purchased from Origene (Rockville, MD). Reverse transient transfections of shRNAs were performed by using Lipofectamine 3000 transfection reagent (#L3000015, Thermo Fisher Scientific). Cells were seeded in 24-well plates (1 × 10^5^ cells/well) and transfected with shRNAs or siRNAs for 48 h before measuring the cell viability with AlamarBlue. Knockdown efficacy of the shRNAs or siRNAs was assessed by quantitative PCR (q-PCR, RT-PCR) and immunoblotting separately.

### Drug combination treatments *in vitro*

*PTEN^+/+^* and *PTEN^-/-^* HCT116 isogenic cells were seeded in 96-well plates for 24 h before adding DMSO control, Anacardic acid, C646, Cycloheximide (CHX), MG132 and MKT077. After incubating with the compounds for 72 h, the cell viability was measured by AlamarBlue assay and were double checked with cell images taken by IncuCyte™ZOOM ((Essen BioScience, Inc., Ann Arbor, Michigan, USA).

### Immunoprecipitation

*PTEN^-/-^* HCT116 cells was treated with 100 μM Anacardic acid for 0, 3, 6 and 9 hours firstly. The cells were then washed with cold PBS once and lysed with the cell lysis buffer containing 20 mM Tris HCl pH 8, 137 mM NaCl, 10% glycerol, 1% Nonidet P-40 (NP-40), 2 mM EDTA and protease inhibitors (1 mM PMSF and a cOmplete protease inhibitor cocktail) on ice for 5 mins. Cell lysates were centrifuged at 12,000 rpm for 5 mins and the supernatant were used for co-immunoprecipitation with an anti-AKT1 antibody, the mouse IgG as the negative control and Dynabeads™ Protein G Immunoprecipitation Kit (Invitrogen#10007D). The immunoprecipitates were subjected to western blotting with the Hsp70, Hsp90 and AKT1 antibodies separately.

### Chromatin Immunoprecipitation

*PTEN^-/-^* HCT116 cells were treated with 100μM Anacardic acid for 9hrs after being seeded. 2.0 × 10^5^ cells were used for each single strip well following the protocol with Imprint Chromatin Immunoprecipitation kit from Sigma-Aldrich (Sigma #CHP1-96RXN). The Ac-H4 antibody (sc-8662-R) was used to immunoprecipitate DNA-protein cross-linked complex while the mouse IgG was used as the negative control. The immunoprecipitated promoter sequences of Hsp70s and Hsp90 were detected with real-time PCR.

### Real-time quantitative PCR (RT-qPCR)

Cells were washed with cold PBS once and lysed in the Real-time cell lysis buffer containing 10mM Tris pH 7.4, 0.25% Igepal CA-630, and 150mM NaCl for 5 mins 18. Cell lysates were directly used for real-time as the amplification templates. All the primers used in RT-qPCR are listed in Supplementary Table S2.

### Overexpression of Hsp70 (*HSPA1A*) and Hsc70 (*HSPA8*)

For overexpression of Hsp70 and Hsc70, HCT116-*PTEN^-/-^*cells (2.0 × 10^5^ cells/well in a 24-well plate) were transfected with pcDNA5/FRT/TO GFP *HSPA1A* (Addgene plasmid, #19483) or pcDNA5/ FRT/TO GFP *HSPA8* (Addgene plasmid, #19487) plasmid mixed in Lipofectamine 3000. The transfected cells were co-treated with or without 100 μM AA for 24 h and the cell viability was measured with AlamarBlue assay.

### *In vivo* drug combination studies on mice xenograft models

Drug effects were checked *in vivo* efficacy on the mice where HCT116- *PTEN^+/+^* and HCT116-*PTEN^-/-^* cells (1 × 10^6^ cells/injection) were injected subcutaneously in each flank (bilaterally) of female Nude mice. After 4 days of tumor cell inoculation, both tumors became palpable. The mice were then randomized into 3 groups (n=5/group) of equal tumor volume for treatments with vehicle alone (DMSO) and Anacardic acid (2.5 mg/kg and 5 mg/kg, solved in DMSO) by subcutaneous injection around the tumor 19. Treatment was done daily for 4 weeks. Tumor size was measured periodically with a vernier caliper and tumor volume was calculated based on the modified ellipsoid formula (long axis × short axis^2^ × π/6). All *in vivo* modelling was carried out in accordance with the approved animal protocol (UMARE-010-2018) by Animal Research Ethics Committee of the University Macau.

### Statistical analysis

The data are represented as the mean ± SD. Statistical analyses were performed using analysis of variance (ANOVA) and Student's t-test with Graphpad Prism. A *P*-value less than 0.05 was considered statistically significant.

## Results

### Small Molecule Screening Identifies Anacardic Acid as Synthetic Lethality Compound in PTEN-Deficient CRC Cells

**Figure 1:**
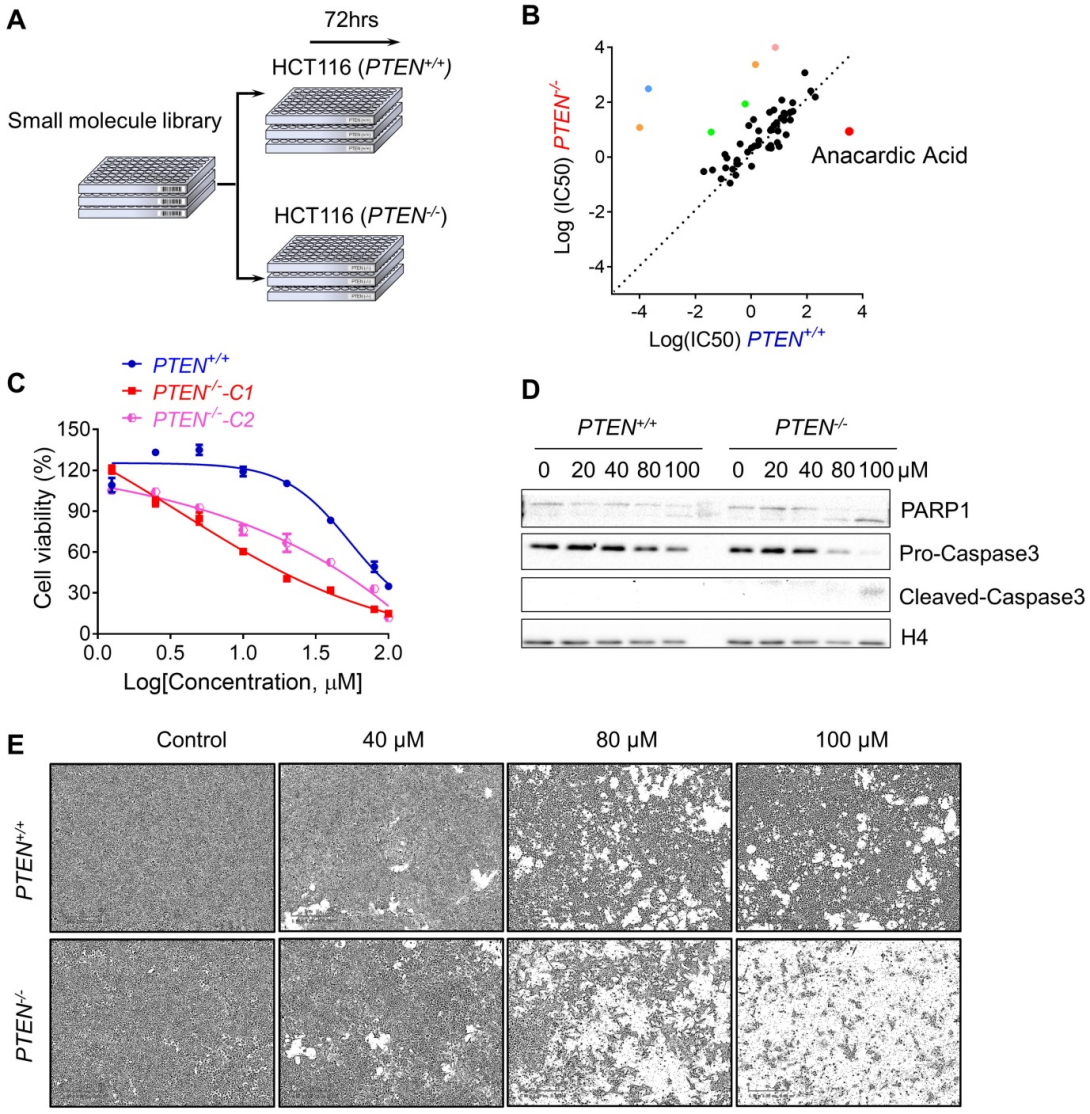
** Synthetic lethality screening with small compound library identified anacardic acid as a synthetic lethality compound in *PTEN^-/-^* colorectal cancer cells. (A)** Schematic representation describing workflow for synthetic lethality screening with small compound library in *PTEN^+/+^* and *PTEN^-/-^* HCT116 cell pairs. **(B)** The log10 plot of the IC50 values of each compound against *PTEN^+/+^* and *PTEN^-/-^* HCT116 cells. Two MEK inhibitors (Trametinib and TAK-733, Green colored), 2 VEGFR inhibitors (Apatinib and Nintedanib, Orange colored), a BTK inhibitor (AVL-290, Blue colored), an SRC inhibitor (PP1, Pink colored) and a p300/CBP inhibitor (Anacardic acid, AA, red colored) are marked as selective inhibitors toward *PTEN^+/+^* or *PTEN^-/-^* HCT116 cells. **(C)** Survival curves of *PTEN^+/+^* and *PTEN^-/-^* HCT116 cells treated with anacardic acid for 72 h. *PTEN^-/-^*-C1 and *PTEN^-/^*^-^-C2 represent *PTEN^-/-^* clone 1 and 2, respectively. **(D)** Western blots of cleaved PARP1 and cleaved caspase 3 in *PTEN^+/+^* and *PTEN^-/-^* HCT116 cells treated with anacardic acid for 72 h. **(E)** Representative cell images from *PTEN^+/+^* and *PTEN^-/-^* HCT116 cells treated with anacardic acid for 72 h.

To perform the synthetic lethality drug screening based on the differences of PTEN status, PTEN isogenic HCT116 pair: *PTEN^+/+^* and *PTEN^-/-^* was used to screen for synthetic lethality compounds^17^. Total 430 small molecules from kinase inhibitor library that targets most of drug-targetable human kinases were screened (Fig. 1A). From a pair-wise, 8-dose titration screening of 430 small molecules, 7 compounds that showed different selectivity in *PTEN^+/+^* cells over the *PTEN^-/-^* part were identified (Fig. 1B). Among the compounds, 2 MEK inhibitors (Trametinib and TAK- 733), 2 VEGFR inhibitors (Apatinib and Nintedanib), a BTK inhibitor (AVL-290) and an SRC inhibitor (PP1) showed the greater selectivity toward *PTEN^+/+^* CRC cells. Anacardic acid (AA), a p300/CBP HAT inhibitor, showed the greater selectivity toward *PTEN^-/-^* CRC cells (Fig. 1B). It is known that PTEN deficiency contributed to the drug resistance to trametinib in M14 melanoma cells 20. Other MEK inhibitors also showed drug resistance with PTEN deletion in acute myeloid leukemia 21. These data demonstrated the feasibility of our synthetic lethality screening system. Since AA was the only compound that showed the selectivity toward *PTEN^-/-^* CRC cells, we studied the effect of AA in greater detail with regard to the PTEN deficiency in CRC cells. AA significantly inhibited the viability of *PTEN^-/-^* cells, while they marginally affected *PTEN^+/+^* cell viability (Fig. 1C and E). Similar results were observed in another *PTEN^-/-^* HCT116 cell line (*PTEN^-/-^*-C2), suggesting that the differential sensitivity of the compound was likely due to the difference in the PTEN status (Fig. 1C). It was further observed that AA significantly induced caspase-3 and PARP1 cleavage in *PTEN^-/-^* cells but not in *PTEN^+/+^*, suggesting that the synthetic lethality effect of AA was via the induction of apoptosis in *PTEN^-/-^* cells (Fig. 1D).

### Inhibition of p300/CBP HAT Activity by shRNA Silencing or Another Small Molecule p300/CBP Inhibitor C646 Recapitulated the Phenotype of Anacardic Acid

**Figure 2:**
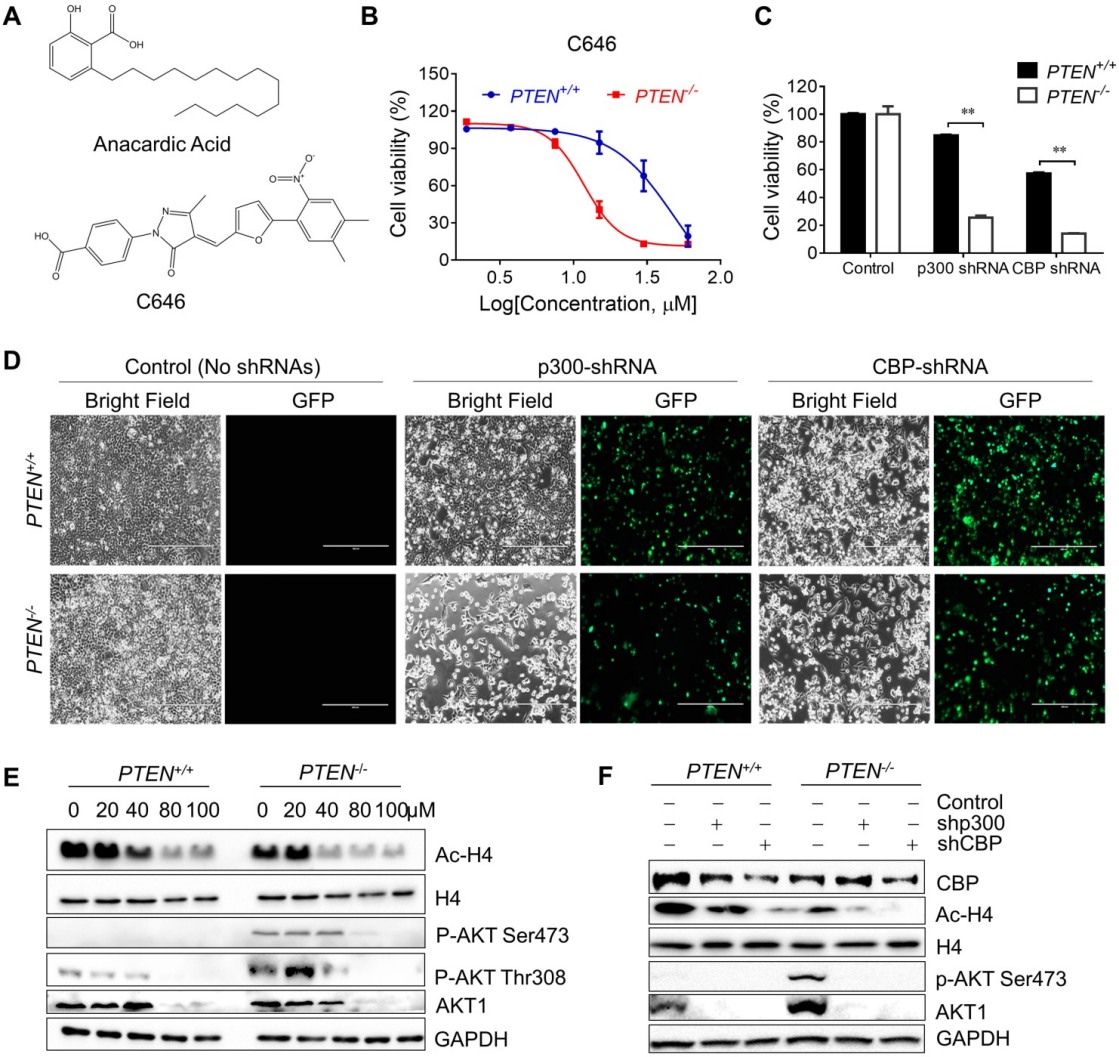
** Inhibiting p300/CBP induced synthetic lethality in *PTEN^-/-^* colorectal cancer cells. (A)** The structures of AA and C646. **(B)** Survival curves of *PTEN^+/+^* and *PTEN^-/-^* HCT116 cells treated with C646. Cells were treated with C646 for 96 h. **(C)** Bar charts of cell viability in *PTEN^+/+^/PTEN^-/-^* cells after transfecting with p300 and CBP shRNAs for 72 h. **(D)** Phase-contrast and fluorescence images of *PTEN^+/+^/PTEN^-/-^* cells transfected with p300 or CBP shRNAs with GFP for 72 h. **(E)** Western blots of Ac-H4, H4, p-AKT Ser473, p-AKT Thr308, AKT1 and GAPDH in PTEN isogenic HCT116 pairs after treated with 0, 20,40, 80, 100 μM anacardic acid for 24 h. **(F)** Western blots of CBP, Ac-H4, p-AKT Ser473, AKT1 and GAPDH after knock-downing p300/CBP with shRNAs for 72 h in PTEN isogeneic HCT116 cell pairs. **P-values ≤ 0.01 in Student's t-test.

Since AA was reported as a HAT inhibitor, we first checked whether the synthetic lethality effect of AA in the *PTEN^-/-^* cells was based on inhibition of HAT activity of p300/CBP or not. The isogenic CRC cells were treated with another specific p300/CBP inhibitor C646 and it was found that *PTEN^-/-^* cells were more sensitive to C646 (Fig. 2B). Then, further exploration was made into the effects of inhibition of p300 and CBP by silencing with specific p300/CBP- EGFP tagged shRNAs separately. Silencing p300 or CBP selectively inhibited the viability of *PTEN^-/-^* HCT116 cells, but not *PTEN^+/+^* ones, indicating that the inhibition of p300 and CBP was likely to mediate the synthetic lethality phenotype induced by AA (Fig. 2C and D; Supplementary Fig. S1). Further analysis of the downstream pathways that might be affected by p300/CBP inhibition or silencing showed that the acetylation of histone H4 decreased while total H4 showed no difference, which was as expected (Fig. 2E and F). These data indicated that AA indeed inhibited the HAT activity of p300 and CBP.

**Figure 3:**
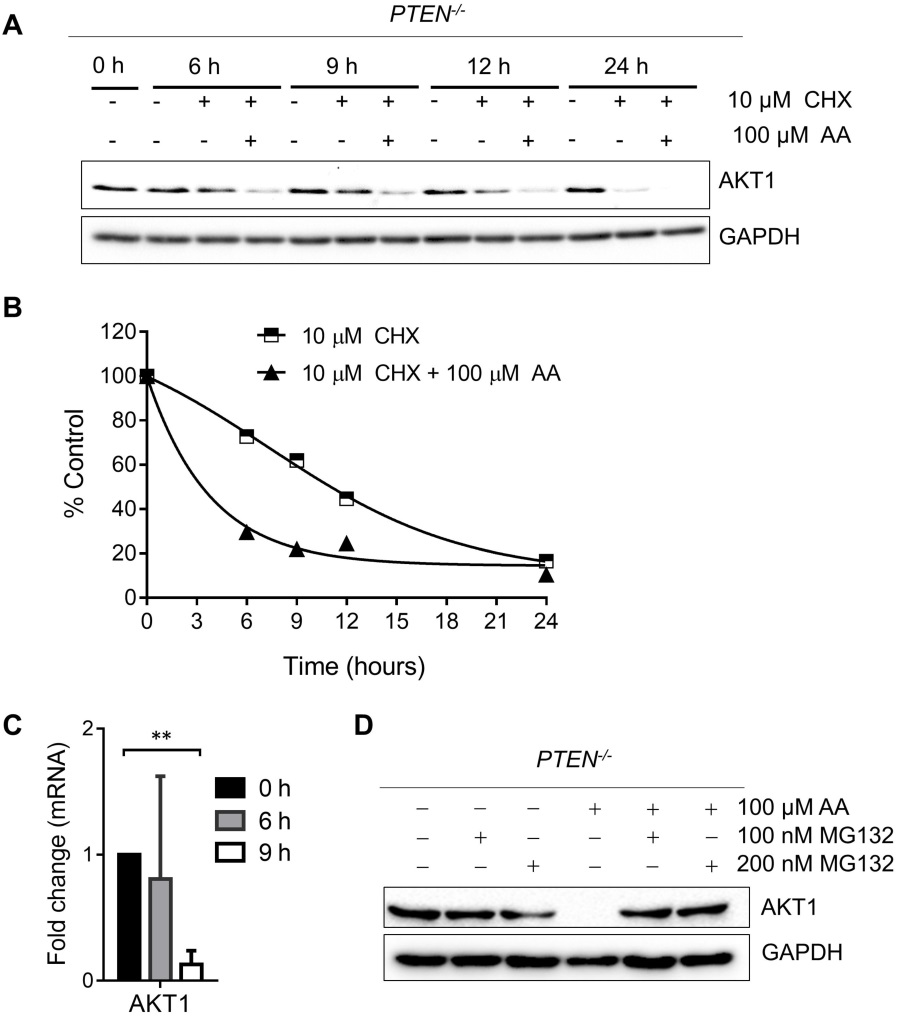
** Anacardic acid reduces the level of AKT at both transcription and post-translational levels. (A)** Western blots of AKT1 in *PTEN^-/-^* cells treated with 10 μM cycloheximide (CHX) combined with or without 100 μM AA for indicated time points. **(B)** Quantification curve of AKT1 protein level based on the western blot of (A). **(C)** RT-qPCR analysis of AKT1 mRNA in *PTEN^-/-^* cells treated with 100 μM anacardic acid for 9 h. **(D)** Western blots of AKT1 and GAPDH in *PTEN^-/-^* cells treated with anacardic acid, MG132 and the combination of both for 24 h. **P-values ≤ 0.01 in Student's t-test.

As PI3K-AKT pathway was well known to be significantly affected by PTEN status, a check on downstream proteins of PTEN was made. The levels of p-AKT-Ser473 and total AKT1 were reduced by AA treatment or the silencing of p300 and CBP (Fig. 2E and F). The reduction of the level of p-AKT-Ser473 was likely to be resulted from the reduction of total AKT level as their reduction dynamics was similar. To test the mechanism how AA reduced the AKT protein level, we treated the *PTEN^-/-^* HCT116 cells with AA alone or combined with the protein translation inhibitor cycloheximide (CHX) for 24 h. Western blotting of AKT1 at 0, 3, 6, 9, 12, 24 h showed that the half-life of AKT1 was around 12 hours after CHX treatment and it was shortened to 3.5 hours when combined with AA (Fig. 3A and B), suggesting that AA significantly reduced the AKT1 protein stability. As acetylation of histones regulates transcription activation and repression 22, we also tested whether AA affect AKT1 transcription. RT-qPCR analysis showed that AA treatment also reduced AKT1 mRNA level (Fig. 3C). In addition, co-treatment of MG132, a proteasome inhibitor, can reverse the effect of AA on the reduction of AKT1 level (Fig. 3D), suggesting that AA reduces AKT1 level at both the transcription and the post-translational levels after inhibition of p300/CBP HAT activity.

### Anacardic Acid Reduces the Transcription of Hsp70 Family by Inhibiting p300/CBP HAT Activity

**Figure 4:**
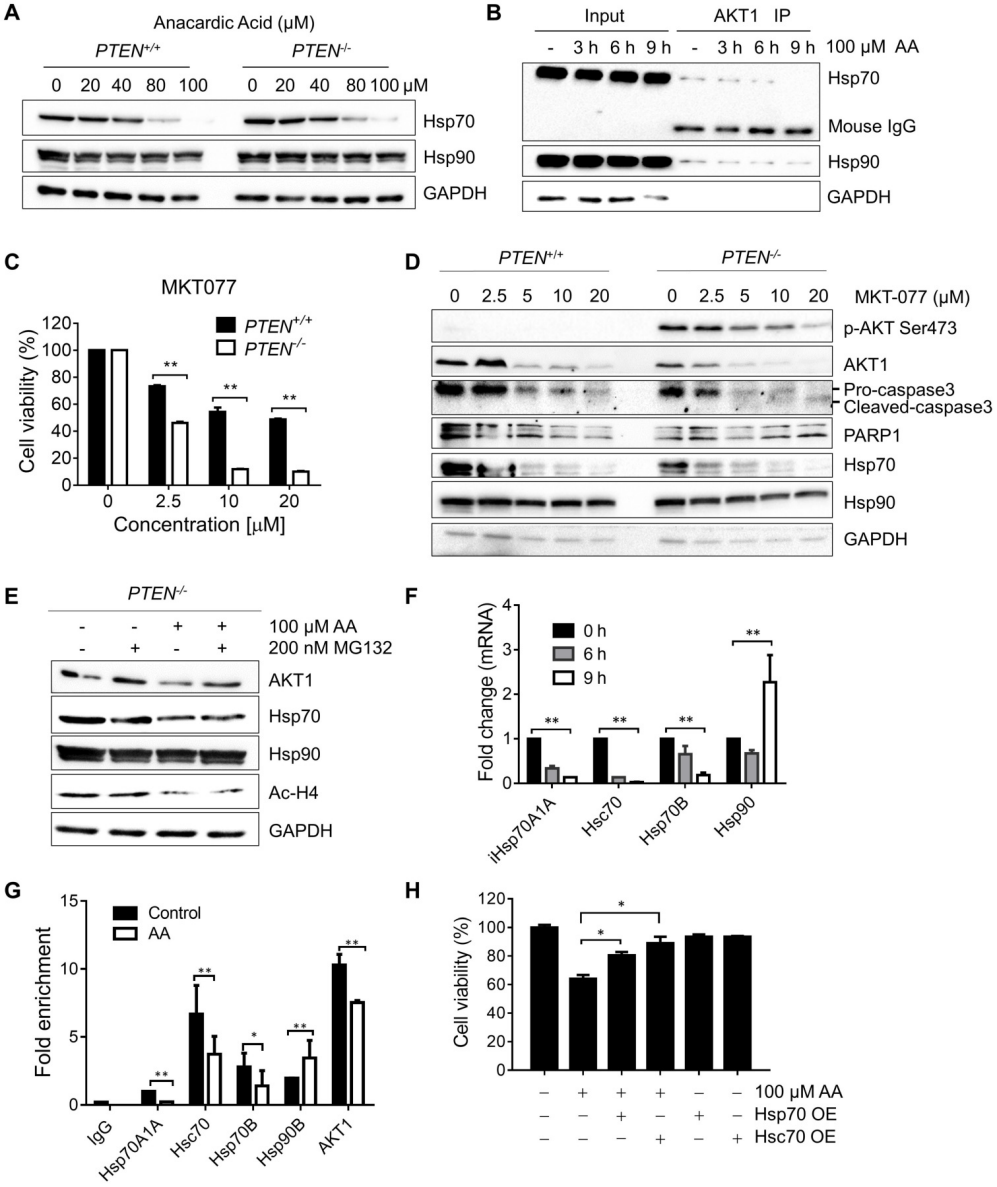
** Anacardic acid decreased the transcription of Hsp70 family by inhibiting P300/CBP HAT activity on promoters of Hsp70 family and induced destabilization of Hsp70/AKT complex. (A)** Western blots of heat shock proteins in PTEN isogenic HCT116 pairs after treated with 0, 20, 40, 80, 100 μM anacardic acid for 24 h. **(B)** Western blots of co-immunoprecipitation experiment of heat shock protein members with AKT1 in *PTEN^-/-^* cells treated with 100 μM AA for 3, 6 and 9 h. **(C)** Bar charts of cell viability after incubating *PTEN^+/+^* and *PTEN^-/-^* HCT116 cell lines with the Hsp70 inhibitor MKT077. **(D)** Western blots of p-AKT Ser473, AKT1, cleaved-caspase3, PARP1, Hsc70, Hsp70, Hsp90 and beta-actin in PTEN isogenic HCT116 pairs after treating with 0, 2.5, 5, 10, 20 μM MKT077 for 24 h. **(E)** Western blots of AKT1, Hsp70, Hsp90, Ac-H4 and GAPDH in *PTEN^-/-^* cells treated with anacardic acid, MG132 and the combination of both for 24 h. **(F)** Real-time qPCR showing the changes in the expression level of Hsp70 and Hsp90 family at 0, 6, 9 h with 100 μM anacardic acid in *PTEN^-/-^* cells. **(G)** Bar charts of immunoprecipitated promoter regions of heat shock protein members and AKT1 with acetylation H4 antibody in ChIP experiments after treating with anacardic acid for 9 h in *PTEN^-/-^* cells. **(H)** The effect of the overexpression of Hsp70/Hsc70 on anti-proliferative effect on AA in* PTEN^-/-^* cancer cells. *PTEN^-/-^* HCT116 cells were transfected with Hsp70 or Hsc70 plasmid and treated with 100 μM AA for 24 h. The cell viability was measured with AlamarBlue assay. *P-values ≤ 0.05; **P-values ≤ 0.01 in Student's t-test.

The heat shock protein 70 (Hsp70s) family works in protein folding, helps misfolded client proteins to refold and degrades the abnormal proteins 23-25. Heat shock protein 90 (Hsp90s) family stabilizes AKT by forming HSP-client complex 24, 26, 27. To explore the mechanisms that underlie the destabilization of AKT and the related decrease of AKT phosphorylation at Ser473, firstly, a check was made on the heat shock proteins which were reported to bind with AKT and form the functional complex 24, 26. Surprisingly, it was found that the protein level of Hsp70, but not Hsp90, was dose-dependently reduced by AA (Fig. 4A). Immunoprecipitation analysis of AKT1/Hsp complex showed that while Hsp70 and Hsp90 proteins co-immunoprecipitated with AKT1, AA significantly reduced the Hsp70 level from the complex (Fig. 4B; Supplementary Fig. S2). These data gave clues that the reduction of Hsp70 level might contribute to the AKT1 destabilization after AA treatment. There are two major cytosolic members of Hsp70 family: the constitutive Hsc70, and the inducible Hsp70 (Hsp70A1A and Hsp70A1B) which are induced under stress and are important for cancer cell survival 28, 29. We thus used MK077, an allosteric inhibitor of Hsp70, which binds to ADP-bound state of Hsp70 and inhibits the release of its substrates 30, to test the effect of Hsp70 inhibition on AKT1 stability and synthetic lethality. Either silencing of the Hsp70 protein family or small molecule inhibitor recapitulated the synthetic lethality phenotype in *PTEN^-/-^* HCT116 cells (Fig. 4C, and D). In order to assess the causal relationship between Hsp70 and AA-induced synthetic lethality, we analyzed the rescue effect of Hsp70 overexpression on the synthetic lethality using two Hsp70 overexpression vectors, including HSPA1A (Hsp70)-GFP and HSPA8 (Hsc70)- GFP (Supplementary Fig. S3). Overexpression Hsp70 or Hsc70 significantly rescued the synthetic lethality effects of AA in *PTEN^-/-^* HCT116 cells (Fig. 4H). These data suggested that the reduction of Hsp70 level was likely the key factor mediating the synthetic lethality effect of AA.

To test whether the reduction of Hsp70 by AA is due to the inhibition at the transcription or post-translational level, the *PTEN^-/-^* HCT116 cells were treated with AA with or without MG132 and Hsp70, Hsp90, AKT and acetyl-H4 levels were analyzed. As a result, MG132 treatment did not reverse the effect of AA on decreasing Hsp70 or acetyl-H4, while it reversed the AKT1 level, indicating that the decrease in Hsp70 level might be due to the inhibition at the transcription level (Fig. 4E). RT-qPCR verified that the mRNAs of Hsp70 family members decreased significantly by AA treatment, while no inhibition on Hsp90 mRNA was observed (Fig.4F). As the acetylation of histones regulates transcription activation and repression 22, further exploration was made in the relationship between the down-regulation of Hsp70 transcription and histone acetylation changes induced by AA. For this, we conducted chromatin immunoprecipitation (ChIP) analysis of the promoter regions of Hsp70 family and Hsp90 genes using anti-acetyl-H4 antibody in *PTEN^-/-^* CRC cells treated with AA. It was found that AA treatment significantly reduced the level of local H4 acetylation on the promoter regions of Hsp70 (Fig. 4G). AA did not affect the H4 acetylation on the promoter region of Hsp90 gene, while it partially reduced the H4 acetylation on the promoter region of AKT1. These data further suggested that AA reduced Hsp70 transcription through the inhibition of p300/CBP HAT activity, and the reduced Hsp70 protein in turn destabilized AKT1 protein level. AA also reduced AKT1 transcription level through the inhibition of HAT activity. The decrease in AKT1 level through transcription and post-translational regulations was likely to make a significant impact on the viability of *PTEN^-/-^* CRC cells.

### Anacardic Acid and C646 Selectively Inhibit PTEN-Deficient Prostate Cancer Cells

**Figure 5:**
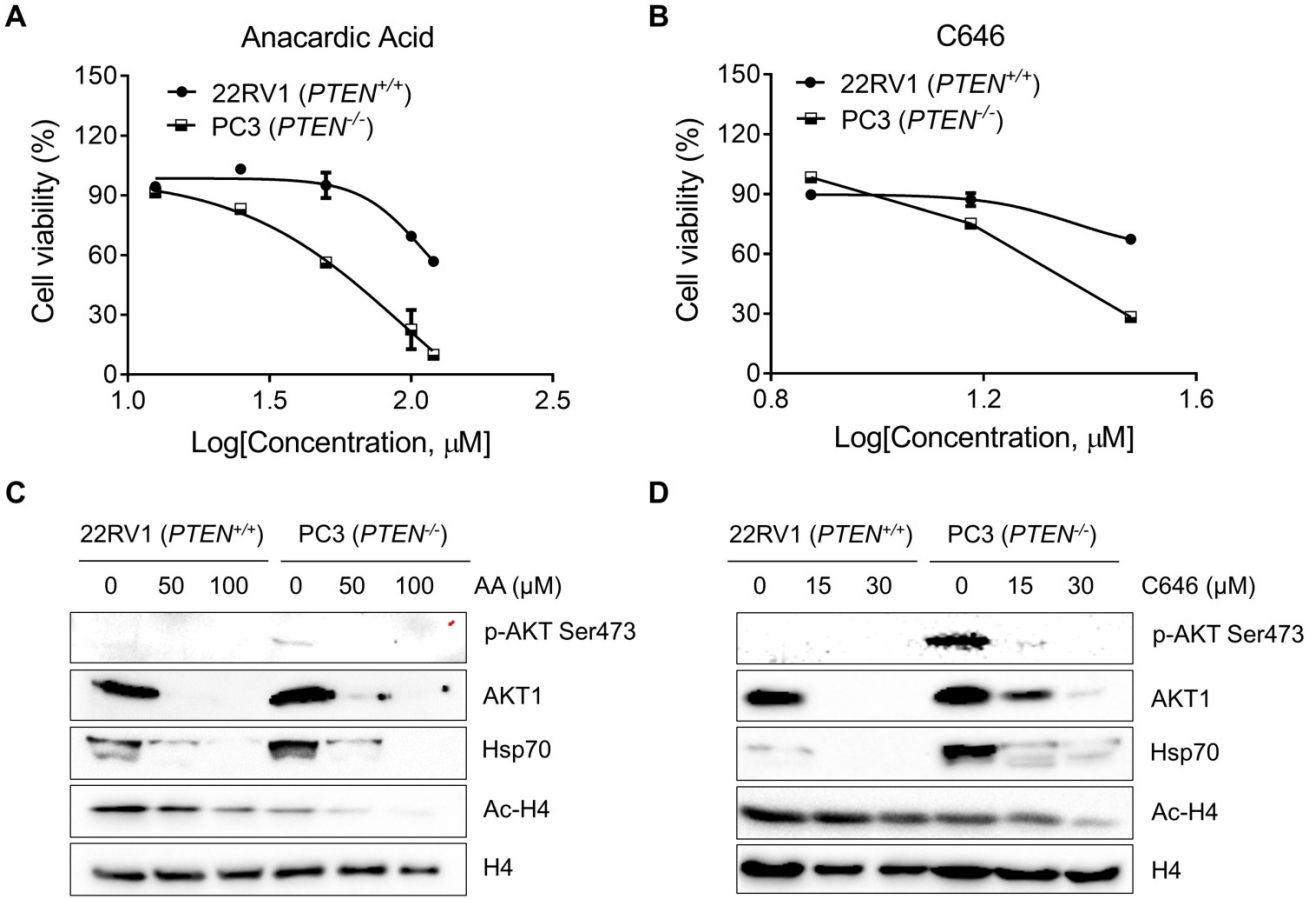
** Anacardic acid and C646 sensitize PTEN deficient prostate cancer cells to die. (A and B)** Survival curves of 22RV1 and PC3 cells treated with AA (A) and C646 (B) for 96 h. **(C and D)** Western blots of p-AKT Ser473, AKT1, Hsp70s, Ac-H4 and H4 in prostate cancer cells treated with two p300/CBP inhibitors, AA (C) and C646 (D) for 24 h.

To further validate that PTEN-mediated synthetic lethality to AA could apply to other cancers; two p300/CBP inhibitors, AA and C646 in a pair of prostate cancer cell lines with different PTEN status: 22RV1 (PTEN wild-type, *PTEN^+/+^*) and PC3 (PTEN null, *PTEN^-/-^*). AA and C646 induced significant cell death in PC3 cells, while they showed marginal effects in 22RV1 (Fig. 5A and B). Hsp70, p-AKT Ser473, AKT1, and Ac-H4 protein levels showed an identical changing trend as what CRC isogenic cell line pair did (Fig. 5C and D). These data further demonstrated that PTEN deficiency resulted in synthetic lethality with the inhibition of HAT activity of p300/CBP in prostate cancer cells.

### Anacardic Acid Showed Synthetic Lethality with PTEN Loss in CRC Mice Xenograft Model

**Figure 6:**
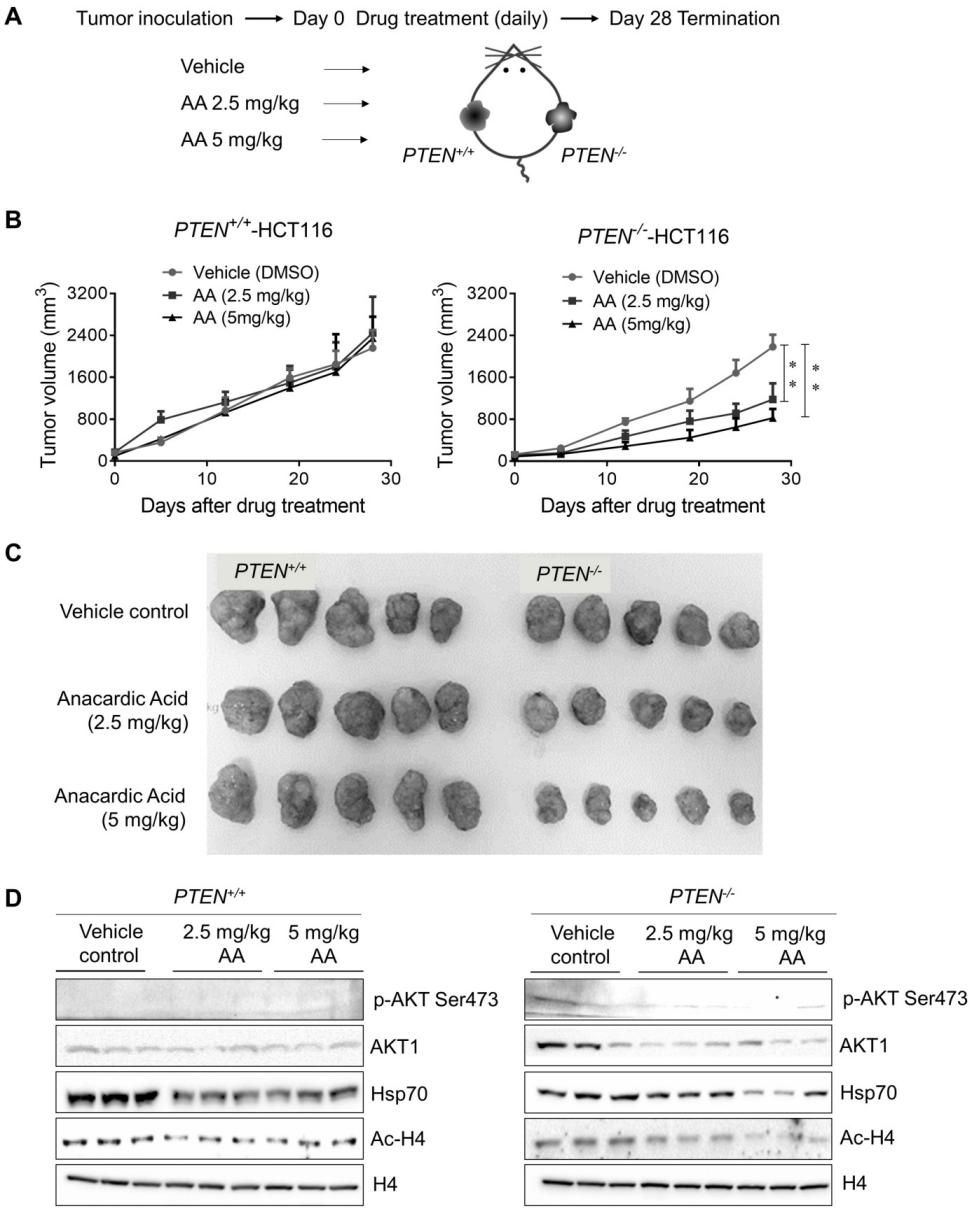
** Xenograft mice models showed anacardic acid reduced tumor growth significantly in *PTEN^-/-^* cancer cells *in vivo*. (A)** Schematic representation of AA drug experiment in mice bearing established *PTEN^+/+^* / *PTEN^-/-^* xenografts. **(B)** Mice were randomized into treatment cohorts of AA (2.5 mg/kg and 5 mg/kg, daily by subcutaneous injection), and vehicle treatments. Mice were treated for a subsequent 28 days' period. Tumor volume was monitored twice weekly. **P-values ≤ 0.01 in ANOVA analysis. **(C)** Whole tumor tissues isolated from mice xenograft cohorts. **(D)** Western blots p-AKT Ser473, total AKT1, Hsp70, Ac-H4 and H4 levels in mice tumor samples.

To further validate PTEN-mediated synthetic lethality to AA, a PTEN-isogenic tumor xenograft mouse model experiment was employed as shown in Fig. 6A. The growth rate of PTEN isogenic CRC cell line pair was identical in 72 *h in vitro* (Supplementary Fig. S4A), which precluded the affection of different growth rates of two cell lines. Mice bearing PTEN- isogenic tumors in both flanks were given AA for 28 days, and tumor volume and mouse body weight were measured periodically. These measurements indicated that all the drug treatment conditions in this study did not show any apparent toxicity in mice (Supplementary Fig. S4B). The treatment with AA significantly delayed the tumor growth of *PTEN^-/-^* HCT116 xenografts (Fig. 6B and C). Whereas the growth of *PTEN^+/+^* HCT116 xenografts was not affected by the same dosage of AA. Similar to that observed in cells, the level of phosphorylated AKT at Ser473 was much higher in *PTEN^-/-^* tumors than in *PTEN^+/+^* ones and was significantly reduced by AA treatment (Fig. 6D). AA treatment also showed the inhibition of histone acetylation at H4 and the reduction of Hsp70 and AKT1 levels in both *PTEN^+/+^* and *PTEN^-/-^* tumors, recapitulating *in vitro* data. These data further demonstrated that the p300/CBP HAT inhibitor AA induced synthetic lethality in PTEN- deficient CRC tumor *in vivo*.

## Discussion

Epigenetic regulation of PTEN-AKT axis occurs commonly in a variety cancers. In Alveolar rhabdomyosarcoma (ARMS) cells, PTEN transcription was repressed by lysine methyltransferase G9a, which activates AKT and promotes the viability of ARMS cells 31. Epigenetic silencing of PTEN was supposed to be related with the inactivation of the tumor suppressor function and the de-repression of AKT pathway in melanoma development 32, 33. Long term treatment of imatinib caused drug resistance by down-regulating PTEN expression with hypermethylation on the promoter of PTEN and activating AKT with the help of highly-enriched DNMT3A and EZH2 in leukemia cells 34, 35. There are also evidences of pathway crosstalk between PTEN and histone acetyltransferases (HATs) in regulating gene transcription. PCAF, a HAT working with p300/CBP in regulating gene transcription, was known to interact and acetylate PTEN, causing down-regulation of PI3K/AKT signaling pathway 36. Loss of PTEN inhibited the activity of Forkhead transcription factors (FOXO) 37. Inactivation of FOXO3a activated p300-dependent hypoxia-inducible factor-1 (HIF-1) transcriptional activity and promoted tumorigenesis subsequently 37. PTEN deficiency facilitated p300 to bind and acetylate androgen receptor (AR) by increasing the phosphorylation of AR in prostate cancer cells. The acetylation of AR by p300 precluded the poly-ubiquitination and hence inhibited proteasome-dependent degradation of AR 38. Knocking down p300 with siRNAs inhibited the proliferation and progression of PTEN-deficient prostate cancer cells and tumors in mice 38. These data suggested the existence of the functional crosstalk between PTEN and HAT via direct interaction or indirectly through regulating epigenetics machinery in cancer.

In this study, from a small molecule screening, we identified the small molecule p300/CBP inhibitor AA as a synthetic lethality compound in PTEN- deficient CRC cells. Mechanistically, AA inhibition of the HAT activity of p300/CBP led to the reduced transcription of Hsp70 family proteins, which in turn caused reduced AKT1-Hsp70 complex formation, inducing AKT1 destabilization. Inhibition of p300/ CBP by AA also reduced the transcription of AKT1, contributing partially to the depletion of AKT1 protein level in AA-treated CRC cells. *PTEN^-/-^* CRC cells have very high level of phosphorylated AKT1 at Ser473 due to the lack of its phosphatase activity, and this high level of phospho-AKT1 makes the cells highly dependent on AKT1 signaling for their survival, a phenomenon called oncogene addiction. Cancer cells with an oncogene addiction are generally hyper-vulnerable to the inhibitors of the oncogene signaling that the cells are highly relying on. Our previous study showed that *PTEN^-/-^* CRC cells were sensitive to AKT inhibition or inhibition of upstream pathways that phosphorylate Ser473 of AKT in CRC 17. *PTEN* deletion or mutation was also reported to increase the sensitivity to AKT inhibitors, PI3K inhibitors, mTOR inhibitors and Hsp90 chaperone inhibitors in breast and other types of cancers 17, 39, 40. Indeed, a number of PI3K/AKT inhibitors are currently under Phase 1 or 2 clinical trials for cancer patients with *PTEN* mutations (https://clinicaltrials.gov/). Therefore, our study suggests that AA-induced depletion of AKT1 protein could be the main factor mediating the synthetic lethality effect in *PTEN^-/-^* CRC cells.

Considering the critical role of AKT activation in PTEN mutated or deleted cancers, eliminating either AKT total protein level or phospho-AKT at Ser473 level would interrupt PI3K-AKT pathway and induce cell death of those cancers that are dependent on AKT. Our study indicate that targeting upstream pathways that activate AKT phosphorylation, or proteins that help stabilizing AKT protein, such as Hsp70s or Hsp90, would be effective strategies for cancer therapy for patients with PTEN loss.

## Supplementary Material

Supplementary figures and tables.
