# Supplementary material for: Histone Acetyltransferase (HAT) P300/CBP Inhibitors Induce Synthetic Lethality in PTEN-Deficient Colorectal Cancer Cells through Destabilizing AKT
Source: Int J Biol Sci. 2020 Mar 25;16(11):1774–84. doi: 10.7150/ijbs.42197 (PMC7211175; doi:10.7150/ijbs.42197)
Supplement: Supplementary file 1 — Supplementary figures and tables. [file ijbsv16p1774s1.pdf]

Supporting Information for

Histone Acetyltransferase (HAT) P300/CBP Inhibitors Induce Synthetic Lethality in PTEN-Deficient Colorectal Cancer Cells through Destabilizing AKT

Yifan Liu, Eun Ju Yang, Changxiang Shi, Pui Kei Mou, Baoyuan Zhang, Changjie Wu, Junfang Lyu, Joong Sup Shim *

Cancer Centre, Faculty of Health Sciences, University of Macau, Taipa, 999078, Macau

*Correspondence: jsshim@um.edu.mo, Tel.: +853-8822-4990

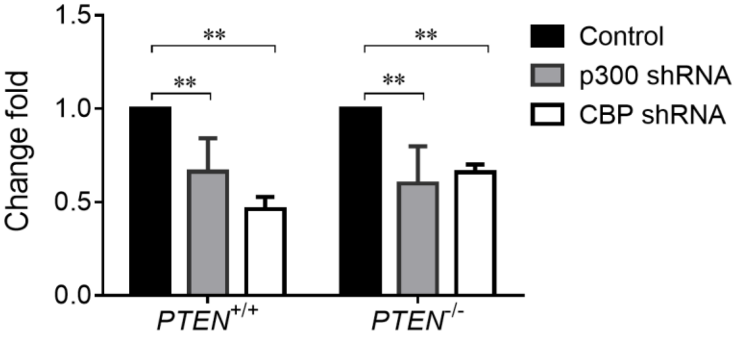

Figure S1. Knockdown efficacy of shRNAs of p300 and CBP. Real-time PCR of p300 and CBP mRNA after transfection with shRNAs for 72h in PTEN$^{+/+}$ and PTEN$^{-/-}$ cells. ** *P*-values ≤ 0.01 in Student's *t*-test.

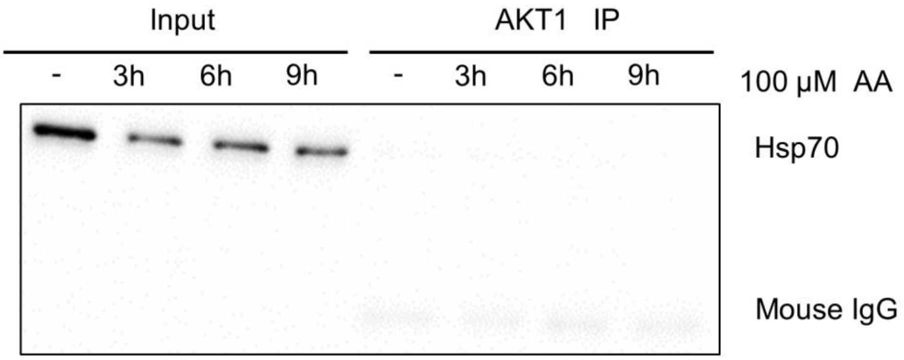

Figure S2. A short exposed image of Hsp70s in immunoblotting. The AA treatment decreased the level of Hsp70 at 3, 6 and 9 h in the input.

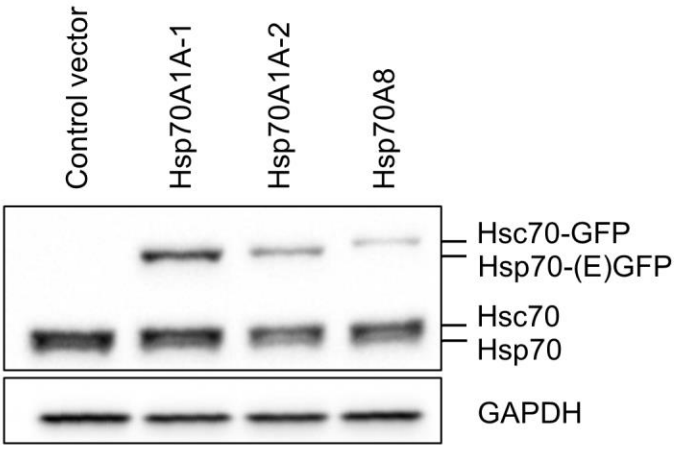

Figure S3. Overexpression of Hsp70s in PTEN$^{-/-}$ cells. The cell lysates were extracted at 24 h after transfected with Hsp70A1A-1 (Hsp70-GFP, Addgene#19483), Hsp70A1A-2 (Hsp70-EGFP, Addgene#15215) and Hsp70A8 (Hsc70-GFP, Addgene#19487) in PTEN$^{-/-}$ cells.

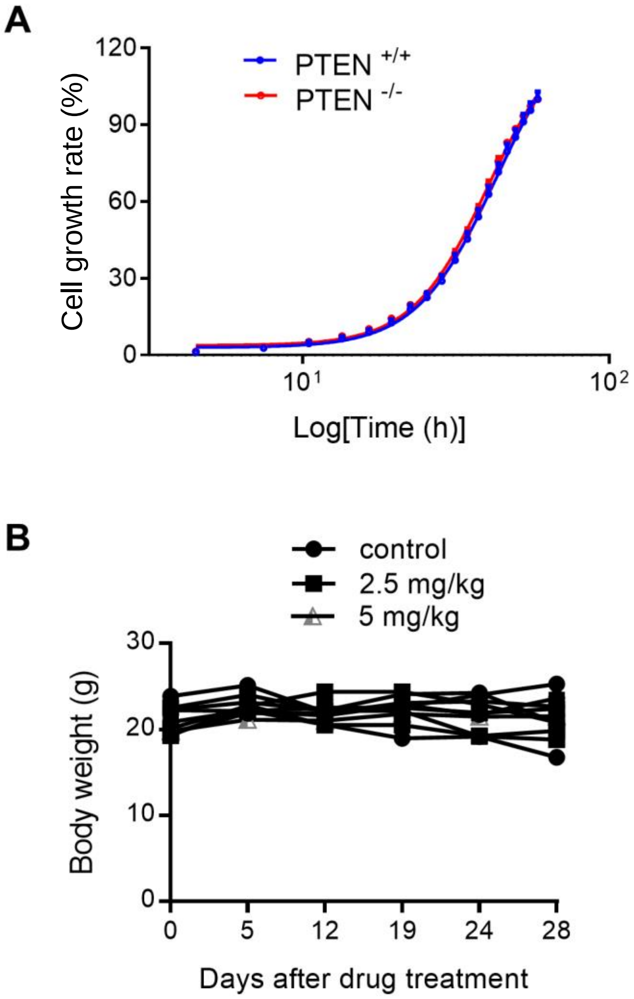

Figure S4. Growth rate of isogenic cell pair and mice body weight changes after drug treatments. (A) Growth rate of $PTEN^{+/+}$ and $PTEN^{-/-}$ cells within 60 h. Data was collected from Incucyte Zoom. (B) Mice body weight of different cohorts (control, 2.5 mg/kg anacardic acid, 5 mg/kg anacardic acid) within 28 days of treatment.

Supplementary Table S1. Primary antibodies used for immunoblotting in the study

**Antibody Name**	**Brand Name**	**Dilution Ratio**
Ac-H4	Santa Cruz, sc-8662-R	1:1,000
AKT1	Santa Cruz, sc-5298	1:1,000
CBP	Cell Signaling, 7389S	1:1,000
Cleaved Caspase-3	Cell Signaling, 9661s	1:1,000
GAPDH	Santa Cruz, sc-365062	1:3,000
H4	Santa Cruz, sc-25260	1:2,000
Hsp70	Santa Cruz, sc-32239	1:2,000
Hsp90	Santa Cruz, sc-69703	1:5,000
p-AKT (Ser473)	Cell Signaling, 9271s	1:1,000
p-AKT (Thr308)	Cell Signaling, 9275s	1:1,000
PARP	Santa Cruz, sc-7150	1:1,000
α-Tubulin	Santa Cruz, sc-5286	1:1,000

Supplementary Table S2. Primers used in quantitative PCR (q-PCR) reaction in this study

**Primer Name**	**Primer sequences**
CREBBP (CBP) Fwd	5'-TGAGAACTTGCTGGACGGAC-3'
CREBBP (CBP) Rev	5'-CTGCCTCCTCGTAGAAGCTC-3'
EP300 (p300) Fwd	5'-GCAGTGTGCCAAACCAGATG-3'
EP300 (p300) Rev	5'-GGGTTTGCCGGGGTACAATA-3'
Hsc70 promoter-Fwd	5'-ATAACAGCTACCTCGCCTGC-3'
Hsc70 promoter--Rev	5'-TATGTGGGGCCTTGTGGTTC-3'
Hsp70A1a promoter-Fwd	5'-GTGTCAACACAAACGCCCC-3'
Hsp70A1a promoter-Rev	5'-GGCGCTGGGCTTTTAATTGT-3'
Hsp70B promoter -Fwd	5'-GGAAGGTGCGGGAAGGTTCG-3'
Hsp70B promoter -Rev	5'-TTCTTGTCGGATGCTGGA-3'
Hsp90β promoter -Fwd	5'-GCGTCTCTCGGACAGGTCAG-3'
Hsp90β promoter -Rev	5'-TCTCGCAGGAGTAGAGGAAGG-3'
HSPA1A-Fwd	5'-AGCTGGAGCAGGTGTGTAAC-3'
HSPA1A-Rev	5'-CAGCAATCTTGGAAAGGCCC-3'
HSPA8-Fwd	5’-TTACCCGTGCCCGATTTGAA-3'
HSPA8-Rev	5’-TTGGCATCTCGAAGGGCTTT-3'
HSPA7-Fwd	5'-CCCTAAGGCTTTCCTCTTGC-3'
HSPA7-Rev	5'-CATGAAGCCGAGCAGTACAA-3'
HSP90B1-Fwd	5’-TTAATCCCAGACACCCGCTG-3'
HSP90B1-Rev	5’-CCGAAGCGTTGCTGTTTCAA-3'
AKT1 Fwd	5'-AGGCACGGGCTAAAGTGAC-3'
AKT1 Rev	5'-CTGTGTGAGCGACTTCATCCT-3'
